# Incidence of Deliberate Self-Harm in Hong Kong Before and During the COVID-19 Pandemic: Population-Wide Retrospective Cohort Study

**DOI:** 10.2196/57500

**Published:** 2025-02-10

**Authors:** Luke Y F Luk, Christie J Y Ching, Tsz Fung Yip, Sunny C L Chan, Catherine Y T Lam, Elizabeth T C Lam, Serena R B Yue, Hoi Ching Pang, Janet Y H Wong, Carlos K H Wong, Chak Kwan Tong, Tafu Yamamoto, Timothy H Rainer, Abraham K C Wai, Joshua W K Ho

**Affiliations:** 1 School of Biomedical Sciences, Li Ka Shing Faculty of Medicine, The University of Hong Kong, Hong Kong SAR, China (Hong Kong); 2 Laboratory of Data Discovery for Health Limited (D24H), Hong Kong Science Park, Hong Kong SAR, China (Hong Kong); 3 Department of Emergency Medicine, Li Ka Shing Faculty of Medicine, The University of Hong Kong, G06, G/F, Jockey Club Building for Interdisciplinary Research, 5 Sassoon Road, Pokfulam, Hong Kong SAR, China (Hong Kong), 852 3917 9175; 4 Li Ka Shing Faculty of Medicine, The University of Hong Kong, Hong Kong SAR, China (Hong Kong); 5 Faculty of Medicine, The Chinese University of Hong Kong, Hong Kong SAR, China (Hong Kong); 6 School of Nursing & Health Sciences, Hong Kong Metropolitan University, Hong Kong SAR, China (Hong Kong); 7 Department of Family Medicine and Primary Care, Li Ka Shing Faculty of Medicine, The University of Hong Kong, Hong Kong SAR, China (Hong Kong); 8 Department of Pharmacology and Pharmacy, Li Ka Shing Faculty of Medicine, The University of Hong Kong, Hong Kong SAR, China (Hong Kong); 9 Intensive Care Unit, Tuen Mun Hospital, Hong Kong SAR, China (Hong Kong); 10 Department of Accident & Emergency, Yan Chai Hospital, Hong Kong SAR, China (Hong Kong); 11 Department of Accident & Emergency, Queen Mary Hospital, Hong Kong SAR, China (Hong Kong); 12 Department of Accident & Emergency, The University of Hong Kong-Shenzhen Hospital, Shenzhen, China

**Keywords:** emergency department, COVID-19, deliberate self-harm, mental health, self-harm, self-injury, self-violence, Hong Kong, SARS-Cov-2

## Abstract

**Background:**

COVID-19 ended on May 5, 2023, and since then Hong Kong reported increased mental distress, which was speculated to be from the policies implemented during the pandemic. Despite this, longitudinal surveillance of deliberate self-harm (DSH) incidences throughout the pandemic in Hong Kong remained insufficient.

**Objective:**

The objective of this study was to outline the changes in DSH incidences before and during the COVID-19 pandemic in Hong Kong, with respect to sex, age, and co-occurring mental health issues.

**Methods:**

A quasi-experiment was conducted using an interrupted time series design to estimate the impact of the pandemic on DSH-related emergency department (ED) visits. This design enabled the estimation of DSH-related ED visits based on prepandemic data from 2016 to 2019, assuming the pandemic had not occurred, and allowed for a comparison with observed DSH-related ED visits during the pandemic. The descriptive results were reported as the observed monthly DSH-related ED visits and observed incidence ratios during the pandemic. Afterwards, a negative binomial model was fitted to the prepandemic data (2016‐2019) and adjusted for temporal trends, seasonality, and population variation to estimate the expected monthly DSH-related ED visits and adjusted incidence ratios (aIRs).

**Results:**

Between January 2016 and December 2022, a total of 31,893 DSH episodes were identified. Initial descriptive analysis showed a significant difference in demographic characteristics (sex) and clinical characteristics (death within 28 d, diagnoses of co-occurring mental health issues, public assistance pay code, and triage level). Subsequent interrupted time-series analysis demonstrated significantly increasing trends in comparison with the prepandemic period. As reported in the aIRs among young adult males (aIR in 2020=1.34, *P*=.002; 2021: aIR=1.94, *P*<.001; and 2022: aIR=2.53, *P*<.001), adult males (aIR in 2020=1.58, *P*<.001; 2021: aIR=2.64, *P*<.001; and 2022: aIR=3.13, *P*<.001), adult females (aIR in 2020=1.13, *P*=.01; 2021: aIR=1.52, *P*<.001; and 2022: aIR=1.64, *P*<.001), and older male adults (aIR in 2020=1.53, *P*<.001; 2021: aIR=2.37, *P*<.001; and 2022: aIR=3.01, *P*<.001).

**Conclusions:**

The average annual DSH-related ED visits increased during the pandemic period. Therefore, there is a need to raise awareness for such vulnerable groups in Hong Kong to prepare for postpandemic spillover.

## Introduction

During the early phase of COVID-19 pandemic, Hong Kong experienced a significant increase in mental health distress within the community [1-3]. Surveys indicated that an alarming 53.9% (299/555) of participants exhibited moderate to very high levels of psychological distress [4]. In response, the Hong Kong Chief Executive allocated HK $300 million (the conversion rate at the latest revision date of the Chief Executive’s 2020 policy address [November 25, 2020] was US $0.129 per unit HKD) to raise mental health awareness [5]. An advisory committee subsequently developed a comprehensive, community-based initiative. This initiative included an education and health promotion program called “Shall We Talk,” emotional support workshop training for school staff, mental health first aid training for service professionals, and the deployment of suicide bereavement liaison officers. Despite these efforts, there were ongoing concerns about the sustainability of these resources [6].

The association between stress and deliberate self-harm (DSH) was well-documented in the literature [7-11]. However, recent local studies on DSH risk factors, conducted before the pandemic, identified factors such as sex, age, social welfare status, and co-occurring mental health disorders (ie, depression, bipolar disorder, personality disorder, and substance misuse) [12]. The literature from other countries on this topic is limited and sometimes contradictory, with mixed definitions of self-harm and varying conclusions about youth as a risk factor [13-17]. This highlights the need for longitudinal surveillance to better understand the pre-existing risk factors of DSH.

One of the major challenges in emergency departments (EDs) is reducing waiting times without compromising the quality of health care, which includes preventing readmission and reducing health care costs. The Hospital Authority in Hong Kong has pledged to limit the waiting time for emergency (triage category 2) patients to 15 minutes, and for urgent (triage category 3) patients to 30 minutes [18]. However, by May 2022, over 1500 patients were still waiting for admissions in ED [19]. This underscores the necessity for effective health policies to manage DSH-related ED visits.

The aim of this study was to describe the impact of COVID-19 on DSH incidences in Hong Kong through a longitudinal analysis of DSH-related ED visits before and during the pandemic. This study was stratified by sex, age, and co-occurring mental health issues. The findings from this study justify the need for further surveillance to monitor a possible spill-over effect post the COVID-19 pandemic.

## Methods

### Study Overview and Settings

This territory-wide cohort study analyzed data from January 2020 to December 2022, which was further categorized into pandemic months (January 2020 to December 2022) and the prepandemic months (January 2016 to December 2019).

### Data Sources and International Classification of Diseases, Ninth Revision, Clinical Modification Codes

Data were sourced from the Clinical Data Analysis and Reporting System (CDARS), an administrative clinical database managed by the Hospital Authority of the Hong Kong Special Administrative Region of China [20], encompassing ED data from 18 public hospitals.

DSH and mental health issues were identified using both nursing codes for self-inflicted injury and the *ICD-9-CM* (*International Classification of Diseases, Ninth Revision, Clinical Modification*) diagnosed by clinicians (Table S1 in Multimedia Appendix 1).

In addition, yearly and midyearly population counts for sex and age groups were obtained from the government in Hong Kong [21].

### Outcome Measures

This study defined DSH according the National Institute for Clinical Excellence guidelines, which describe it as “self-poisoning or injury, irrespective of the apparent purpose of the act” [22]. This guideline was adopted due to the absence of patients’ intent in the medical records from CDARS.

Other key definitions included age groups, public assistance pay codes, triage categories, and mental health issues. Age was categorized into teenagers (12‐17 y), young adults (18‐24 y), adults (25‐64 y), and older adults (65‐84 y). Public assistance pay codes referred to the recipients of the Hospital Authority medical fee waiver schemes (Residential Care Service Voucher, Comprehensive Social Security Assistance, and Old Age Living Allowance) [23]. Triage categories 1 to 3 were defined in Table S2 in Multimedia Appendix 1 [18]. Mental health issues included mood disorders, neurosis, alcohol and drug-associated mental health conditions, and other psychotic illnesses and developmental disorders. These diagnoses were identified by *ICD-9-CM* codes, as defined in Table S1 in Multimedia Appendix 1.

### Data Analysis

First, a descriptive analysis of the outcome measures was conducted to identify statistical significance before and during COVID-19. Fisher exact and Mann-Whitney *U* tests were used to compare DSH incidences, sex, age groups, and co-occurring mental health issues.

Next, a quasi-experiment was conducted using an interrupted time series (ITS) design to estimate the impact of the COVID-19 pandemic on DSH-related ED visits. Quasi-experiments estimate causal relationships between different factors and outcomes when random assignment is not possible. The ITS design enabled the estimation of DSH-related ED visits during the pandemic period (2020 to 2022) based on prepandemic data (2016 to 2019). This allowed for a comparison between the observed DSH-related ED visits and the expected DSH-related ED visits in the absence of the pandemic.

Segmented regression is a method commonly used in ITS, typically involves assuming linear trends and identifying significant break points. Given the complexity of COVID-19’s impact on self-harm ED visits, a nonsegmented regression model was chosen, fitted with prepandemic data instead of a segmented-regression model fitted with all data. Self-harm–related ED visits were aggregated into monthly counts to ITS analysis for each group. This analysis was done by fitting prepandemic data to the negative binomial models, with a linear term for trend, and optionally with Fourier terms to account for seasonality, using population terms as an offset. The candidate models can be described by:


$$E\left[y\right]=\exp\left(\beta_{0}+\beta_{trend}\cdot x_{t}+\sum_{k=1}^{n}\left[\beta_{1,k}\cdot \sin\left(\frac{2\pi kx_{t}}{12}\right)+\beta_{2,k}\cdot \cos\left(\frac{2\pi kx_{t}}{12}\right)\right]\right)\cdot population$$

Or


$$E\left[y\right]=\exp\left(\beta_{0}+\beta_{trend}\cdot x_{t}\right)\cdot population$$

The dependent variable y represents the monthly count of self-harm incidents for a particular subgroup. Where $x_{t}$ is the number of months from the start of the study period, n is the highest degree of Fourier terms, $\beta_{0}$$\beta_{trend}$ is the prepandemic slope of incidence rate, $\beta_{1,k}$$\beta_{2,k}$ are coefficients for seasonality. The inclusion of the Fourier term and its degree n were guided by the Akaike Information Criterion (AIC). The final model is the one with the minimal AIC value, ensuring the best balance between model fit and complexity.

The findings from this study were presented as observed incidence ratio (oIR) and adjusted incidence ratio (aIR). The respective formulas were as follows:


$$oIR=\;\frac{Observed\;annual\;DSH\;related\;ED\;visits}{Prepandemic\;annual\;DSH\;related\;ED\;visits}$$

$$aIR=\;\frac{Observed\;annual\;DSH\;related\;ED\;visit\;rate}{Expected\;annual\;DSH\;related\;ED\;visit\;rate}$$

In the formulas, the expected annual DSH-related ED visits were estimated using negative binomial models and accounted for trends, seasonal variations, and population changes. Annual counts were estimated by summing monthly counts, assuming the population remained constant. The aIR compares the observed number of DSH-related ED visits during the pandemic to the expected number based on prepandemic data, providing an estimate of the COVID-19 pandemic’s impact on DSH-related ED visits. Time series graphs were then generated to illustrate these trends. All *P* values less than .05 were considered statistically significant. All statistical analyses were performed using RStudio (version 4.1.0; R Foundation for Statistical Computing).

### Ethical Considerations

The study protocol received approval from the institutional review board of the Hospital Authority, Hong Kong West Cluster (reference UW 20‐112; Multimedia Appendix 2). Informed consent was waivered, and all the data were anonymized.

## Results

### Overview

**Figure 1.**
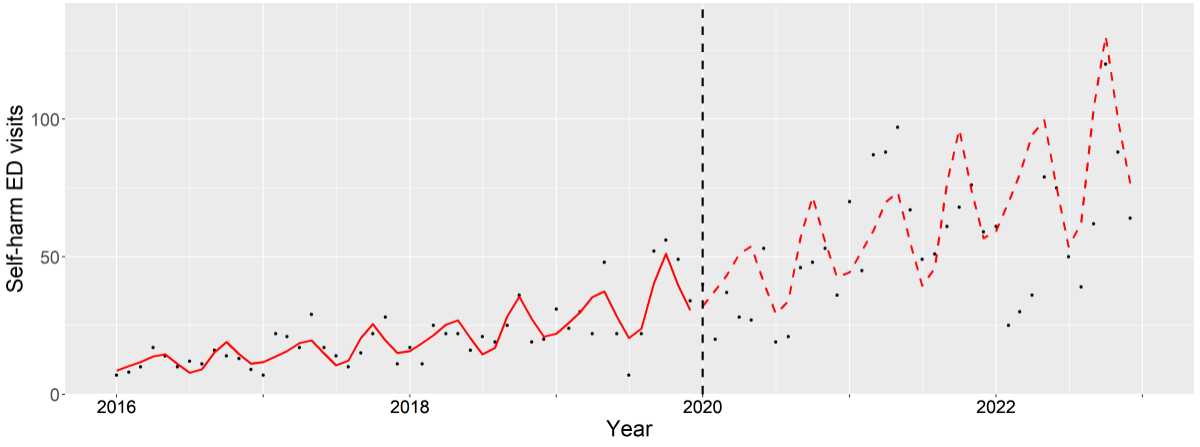
Subgroup analysis of female teenagers (12‐17 years old). The observed (black dots) and expected (red line) number of deliberate self-harm–related emergency department visits between 2016 and 2022. The vertical black dash line indicates the beginning of pandemic period (January 2020). ED: emergency department.

**Figure 2.**
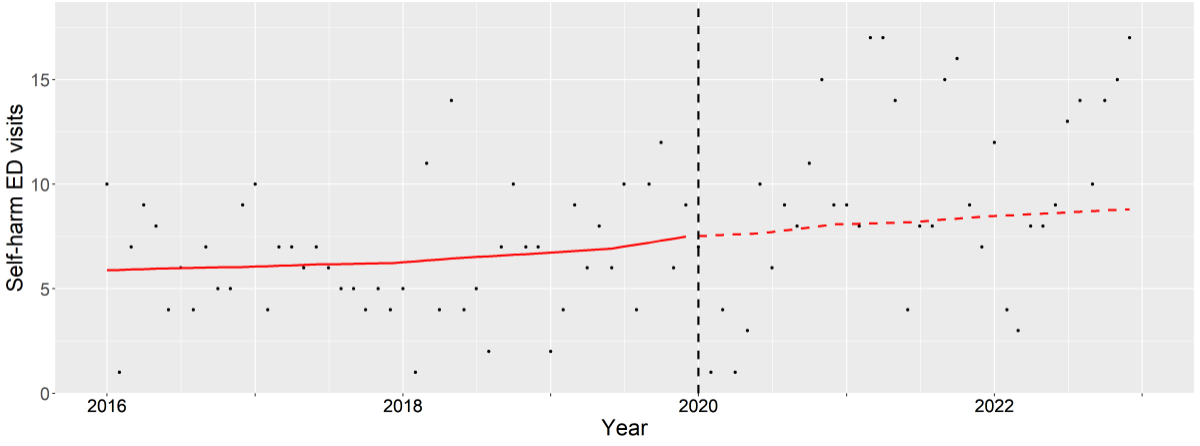
Subgroup analysis of male teenagers (12‐17 years old). The observed (black dots) and expected (red line) number of deliberate self-harm–related emergency department visits between 2016 and 2022. The vertical black dash line indicates the beginning of pandemic period (January 2020). ED: emergency department.

**Figure 3.**
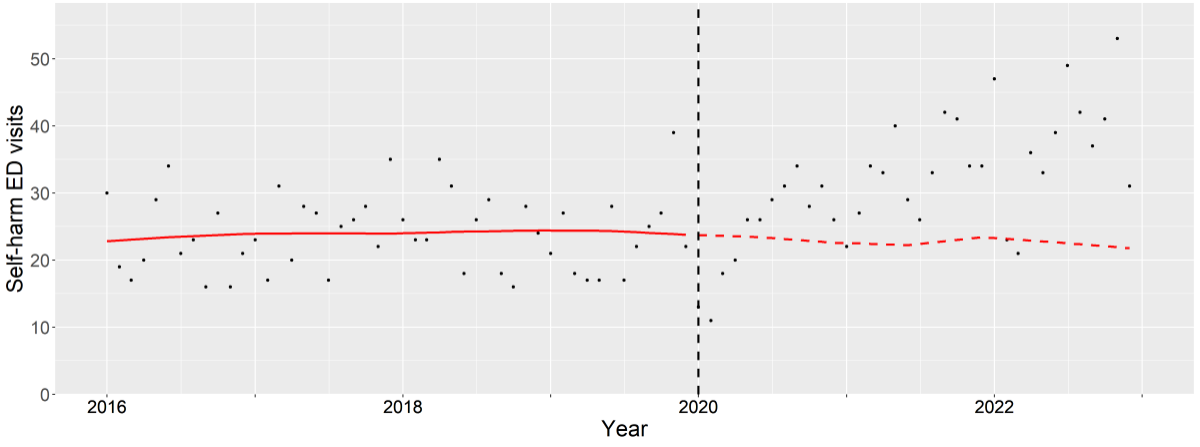
Subgroup analysis of female young adults (18‐24 years old). The observed (black dots) and expected (red line) number of deliberate self-harm–related emergency department visits between 2016 and 2022. The vertical black dash line indicates the beginning of pandemic period (January 2020). ED: emergency department.

**Figure 4.**
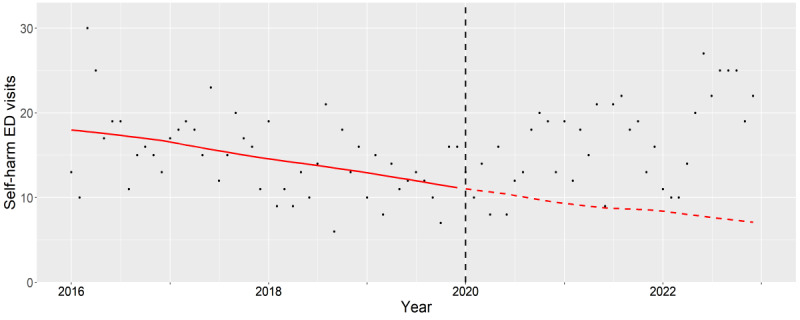
Subgroup analysis of male young adults (18‐24 years old). The observed (black dots) and expected (red line) number of deliberate self-harm–related emergency department visits between 2016 and 2022. The vertical black dash line indicates the beginning of pandemic period (January 2020). ED: emergency department.

**Figure 5.**
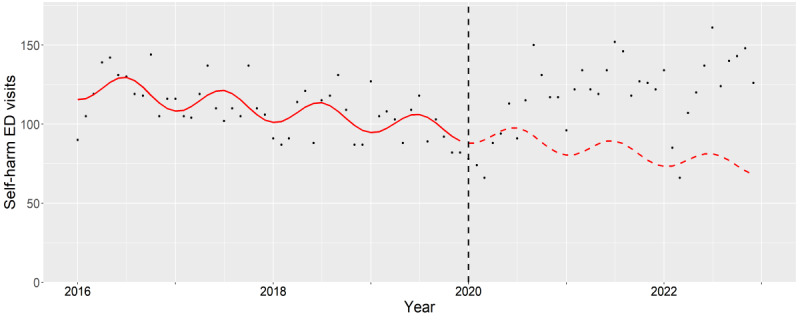
Subgroup analysis of female adults (25‐64 years old). The observed (black dots) and expected (red line) number of deliberate self-harm–related emergency department visits between 2016 and 2022. The vertical black dash line indicates the beginning of pandemic period (January 2020). ED: emergency department.

**Figure 6.**
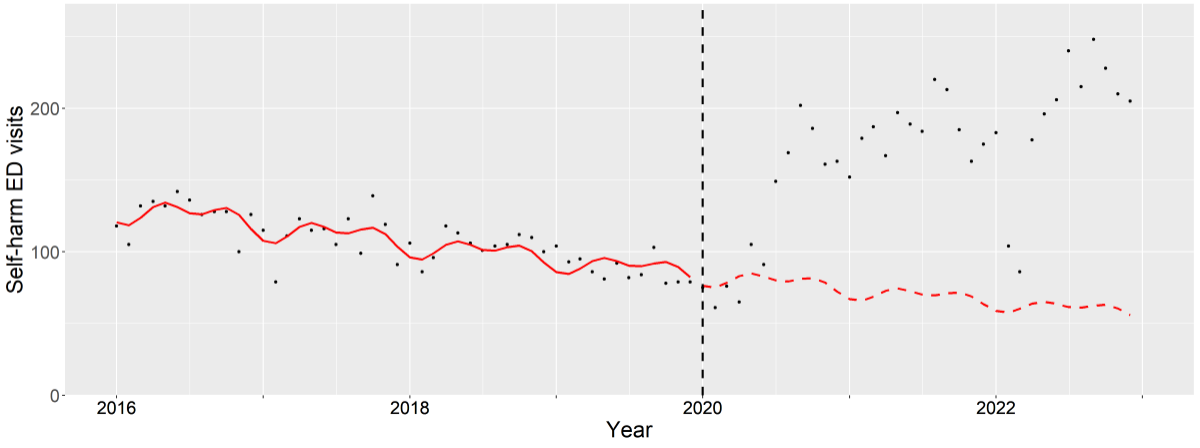
Subgroup analysis of male adults (25‐64 years old). The observed (black dots) and expected (red line) number of deliberate self-harm–related emergency department visits between 2016 and 2022. The vertical black dash line indicates the beginning of pandemic period (January 2020).

**Figure 7.**
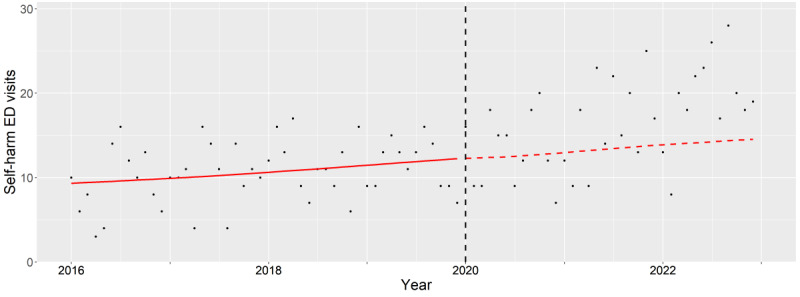
Subgroup analysis of female older adults (65‐84 years old). The observed (black dots) and expected (red line) number of deliberate self-harm–related emergency department visits between 2016 and 2022. The vertical black dash line indicates the beginning of pandemic period (January 2020). ED: emergency department.

**Figure 8.**
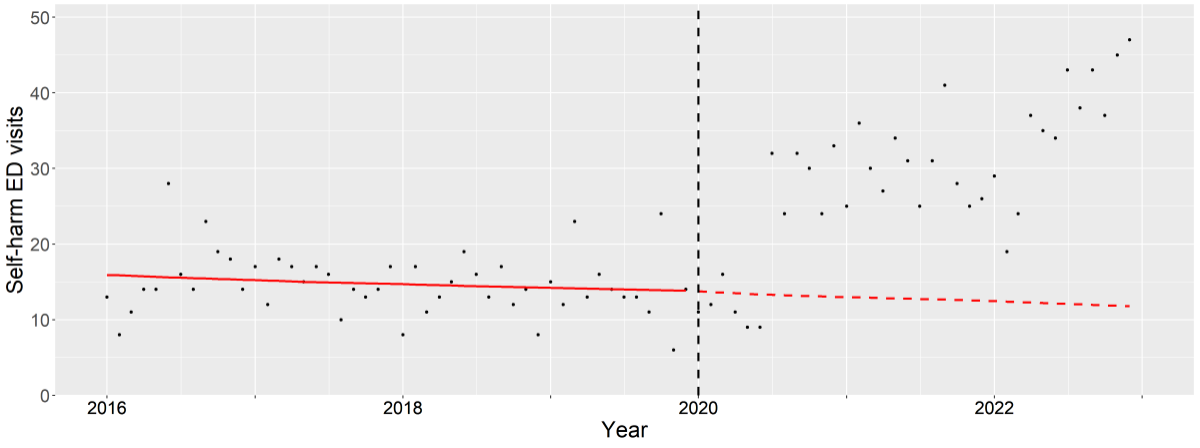
Subgroup analysis of male older adults (65‐84 years old). The observed (black dots) and expected (red line) number of deliberate self-harm–related emergency department visits between 2016 and 2022. The vertical black dash line indicates the beginning of pandemic period (January 2020). ED: emergency department.

A total of 31,893 cases of DSH-related ED visits were identified during the study period. Initial descriptive analysis compared demographic characteristics between the prepandemic and pandemic periods (Table 1). The total number of ED visits with DSH increased by 1175 out of 15,359 (7.7%) patients. There were significantly higher DSH-related ED visits in males (*P*<.001), co-occurring mental health issues diagnosis (*P*<.001), triage category 3 (urgent), and 4 (semiurgent) (*P*<.001) during the pandemic. Conversely, there were significantly less deaths within 28 days (*P*<.001) and less patients with public assistance pay code during the pandemic (*P*=.046). These trends were further explored with subgroup analysis using ITS models (Table 2, Figures 1-8). Further subgroup analysis of co-occurring mental health issues diagnoses were explored in Table S2 in Multimedia Appendix 1.

**Table 1.** Demographic characteristics in patients with deliberate self-harm who visited the emergency department before and during the pandemic (total N=31,893).

Characteristic		Time period		*P* value
		Prepandemic (n=15,359)	Pandemic (n=16,534)	
Age (years), median (IQR)		38 (25)	39 (30)	.60^a^
**Sex, n (%)**				<.001^b^
	Female	8184 (53)	8258 (50)	
	Male	7175 (47)	8276 (50)	
**Deaths within 28 days, n (%)**				<.001^b^
	No	15,102 (98)	16,352 (99)	
	Yes	257 (2)	182 (1)	
**Co-occurring mental health issues diagnosis, n (%)**				<.001^b^
	No	5991 (39)	4878 (30)	
	Yes	9368 (61)	11,656 (71)	
**Public assistance pay code, n (%)**				.046 ^b^
	No	12,060 (79)	13,134 (79)	
	Yes	3299 (22)	3400 (21)	
**Triage, n (%)**				<.001^a^
	Category 1 (critical)	1885 (12)	1329 (8)	
	Category 2 (emergent)	5944 (39)	5450 (33)	
	Category 3 (urgent)	6429 (42)	8539 (52)	
	Category 4 (semiurgent)	1055 (7)	1169 (7)	
	Category 5 (nonurgent)	29 (0)	30 (0)	

a Mann-Whitney *U* test.

b Fisher exact test.

**Table 2.** The number of average yearly visits, observed incidence ratio, and adjusted incidence ratio in deliberate self-harm–related emergency department visits between 2016 and 2022.

Patient characteristic		Years									
		2016‐2019	2020			2021			2022		
		Average yearly visits,n (%)	oIR^a^	aIR^b^	*P* value	oIR	aIR	*P* value	oIR	aIR	*P* value
**Female teenager (12‐17 years old)**											
	Total	251 (100)	1.71	0.78	.98	3.26	1.10	.27	2.90	0.73	.95
	All mental health issues	156 (62)	1.89	0.86	.86	3.99	1.34	.06	3.31	0.82	.81
	Alcohol and drug associated mental health conditions	14 (6)	2.69	1.50	.11	4.58	2.07	.05	3.78	1.39	.28
**Male teenager (12‐17 years old)**											
	Total	77 (100)	1.09	0.92	.70	1.71	1.36	.07	1.65	1.25	.21
	All mental health issues	37 (48)	1.47	1.07	.35	2.67	1.73	.04	2.50	1.47	.15
	Alcohol and drug associated mental health conditions	6 (8)	2.24	2.46	.01	3.04	3.52	.01	4.16	5.13	.02
**Female young adult (18‐24 years old)**											
	Total	287 (100)	1.02	1.06	.25	1.38	1.48	.002	1.57	1.71	<.001
	All mental health issues	149 (52)	1.21	1.24	.04	1.53	1.60	.005	1.83	1.91	.004
	Alcohol and drug associated mental health conditions	47 (16)	1.16	1.64	.008	1.61	2.65	.001	1.91	3.58	.001
**Male young adult (18‐24 years old)**											
	Total	177 (100)	0.93	1.34	.002	1.15	1.94	<.001	1.30	2.53	<.001
	All mental health issues	107 (61)	0.96	1.48	.01	1.37	2.53	<.001	1.61	3.51	<.001
	Alcohol and drug associated mental health conditions	66 (37)	1.08	2.04	.001	1.68	4.08	<.001	2.08	6.38	<.001
**Female adult (25‐64 years old)**											
	Total	1316 (100)	0.94	1.13	.01	1.15	1.52	<.001	1.13	1.64	<.001
	All mental health issues	765 (58)	0.96	1.27	<.001	1.26	1.89	<.001	1.23	1.23	<.001
	Alcohol and drug associated mental health conditions	320 (24)	1.20	1.88	<.001	1.86	3.55	<.001	1.88	4.35	<.001
**Male adult (25‐64 years old)**											
	Total	1289 (100)	1.17	1.58	<.001	1.72	2.64	<.001	1.78	3.13	<.001
	All mental health issues	915 (71)	1.24	1.83	<.001	2.00	3.49	<.001	2.09	4.28	<.001
	Alcohol and drug associated mental health conditions	713 (55)	1.35	2.07	<.001	2.32	4.27	<.001	2.39	5.26	<.001
**Female older adult (65‐84 years old)**											
	Total	128 (100)	1.25	1.04	.33	1.54	1.19	.15	1.81	1.32	.08
	All mental health issues	59 (46)	1.27	1.27	.09	1.64	1.63	.02	2.14	2.13	.002
	Alcohol and drug associated mental health conditions	14 (11)	1.85	1.29	.23	3.78	2.27	.07	3.93	2.05	.16
**Male older adult (65‐84 years old)**											
	Total	177 (100)	1.37	1.53	<.001	2.03	2.37	<.001	2.43	3.01	<.001
	All mental health issues	104 (59)	1.60	1.71	<.001	2.57	2.84	<.001	3.19	3.66	<.001
	Alcohol and drug associated mental health conditions	70 (40)	1.92	2.11	<.001	3.35	3.83	<.001	4.10	4.93	<.001

a oIR: observed incidence ratio.

b aIR: adjusted incidence ratio.

### Teenagers

For teenage females, the oIRs and aIR showed no difference (2020: *P*=.98, 2021: *P*=.27, and 2022: *P*=.95), as reflected by the overlap of the expected and observed trends (Figure 1).

For teenage males, the aIRs showed no difference (2020: *P*=.70, 2021: *P*=.07, and 2022: *P*=.21), as highlighted by the overlap of the observed and expected trends during the study period (Figure 2). However, the aIRs among those with alcohol and drug-associated mental health conditions were significantly different and increased during the pandemic (2020: *P*=.01, 2021: *P*=.01, and 2022: *P*=.02).

### Young Adults

For female young adults, the observed trend was higher than expected during the pandemic (Figure 3). The highest aIR were observed among those with alcohol and drug-associated mental health conditions, with significant difference (2020: *P*=.008, 2021: *P*=.001, and 2022: *P*=.001).

For male young adults, the aIRs between 2020 and 2022 all significantly increased during the pandemic (2020: *P*=.002, 2021: *P*<.001, and 2022: *P*<.001). Further illustrated when the observed trend was higher than the expected trend during the pandemic (Figure 4). Those with alcohol and drug associated mental health conditions had the highest aIRs with statistical significance (2020: *P*=.001, 2021: *P*<.001, and 2022: *P*<.001).

### Adults

The aIRs among adult females significantly increased during the pandemic (2020: *P*=.01, 2021: *P*<.001, and 2022: *P*<.001). This was highlighted when the expected trend decreased while the observed trend increased during the pandemic (Figure 5). The highest aIRs were seen in patients with co-occurring alcohol and drug associated mental health conditions with statistical significance (2020: *P*<.001, 2021: *P*<.001, and 2022: *P*<.001).

For adult males, the aIRs significantly increased during the pandemic (2020: *P*<.001, 2021: *P*<.001, and 2022: *P*<.001). As illustrated when the expected trend decreased while the observed trend increased during the pandemic (Figure 6). Significantly highest aIRs were observed among those with co-occurring alcohol and drug associated mental health condition (2020: *P*<.001, 2021: *P*<.001, and 2022: *P*<.001).

### Older Adults

During 2021 and 2022, older female adults with mental health issues exhibited significantly higher aIRs (2021: *P*=.02, 2022: *P*=.002). These rates were elevated compared with the expected levels during 2021 and 2022 but declined during 2020 (Figure 7).

For older male adults, the aIRs significantly increased throughout the pandemic period (2020: *P*<.001, 2021: *P*<.001, and 2022: *P*<.001). Further underscored by the divergence between the decreasing expected rates and the increasing observed rates during the pandemic (Figure 8). The most pronounced aIRs with statistical significance were among found in older male adults with co-occurring alcohol and drug associated mental health conditions (2020: *P*<.001, 2021: *P*<.001, and 2022: *P*<.001).

## Discussion

### Principal Results

The findings from this study revealed differences in DSH incidences before and during the pandemic, with a notable rise among males and individuals with co-occurring alcohol and drug-related mental health issues.

Previous studies conducted before the pandemic also reported higher rates of DSH among males, which contrasts with the trends observed during our study period [12,24]. This discrepancy may be influenced by cultural factors [12]. For instance, past research has shown that while Chinese males in Hong Kong demonstrate health-seeking behavior, they often hesitate to consult doctors, preferring over-the-counter medications instead [25]. Such tendencies could be exacerbated by the mental distress linked to the isolation policies, as noted in another Hong Kong-based study [26]. However, further research is essential to elucidate the impact of COVID-19 policies on the rising incidences of male DSH in Hong Kong.

In addition, the increased incidences of DSH among individuals with alcohol and drug-related mental health issues observed in this study align with local government reports. The Narcotics Division of the Security Bureau in Hong Kong reported a surge in the use of cocaine, cannabis, ketamine, and heroin combined with methamphetamine during the pandemic [27]. In April 2020, the Centre of Health Protection in Hong Kong also noted a 5.5% increase in alcoholic consumption since the outbreak [28]. This increase in substance use may have been fueled by misinformation circulated on social media, suggesting that alcohol or smoking could protect against COVID-19 [29]. Despite the well-established link between self-harm and substance misuse [30], further research is needed to determine the association between these behaviors and COVID-19 policies.

Several additional patterns emerged from this study. First, there was a decline in DSH-related ED visits in early 2020 and 2022 as well as peaking staying times (Multimedia Appendices 3-6), consistent with the findings from both local and international studies [31-33]. This decline may be attributed to the stigma surrounding mental health issues, which can complicate health-seeking behavior, delay necessary care, and result in more severe DSH presentation requiring readmission to the ED [32,34]. Second, a significant decrease in patients requiring social security suggests that patients performing DSH may come from higher socioeconomic backgrounds. However, socioeconomic profiles varied across epidemic waves [35], indicating the need for further research to validate these findings. Finally, patients performing DSH might have been triaged to less urgent categories due to the changes in ED policies aimed at managing overcrowded cases of infection. These additional findings suggest that patients performing DSH had reduced access to health care services during COVID-19, potentially leading to worsening conditions and long-term repercussions postpandemic.

Given the empirical evidence from this study and existing literature, continued surveillance post the COVID-19 pandemic is essential to determine whether a spillover effect occurred. This need is particularly pressing in light of recent changes in the Hong Kong government’s policies such as the announcement of a liquor tax cut to promote trade in 2024 [36]. Ongoing research and surveillance will be crucial in developing targeted interventions, guiding policy-making, and enhancing mental health services. By closely monitoring these trends, we can better address the mental health challenges posed by the pandemic and its aftermath, ultimately improving outcomes for vulnerable populations.

### Strengths and Limitations

This study has several strengths and limitations. A major strength was the large sample size, which enhances reliability of the findings. In addition, the trend, seasonal, and population factors were adjusted for in the ITS analysis. However, there are notable limitations as well. The absence of data from outpatient and private clinics may reduce the generalizability of our results. Furthermore, the use of *ICD-9-CM* codes to identify DSH cases could compromise the accuracy of our findings, as later versions of the *ICD* (*International Classification of Diseases*) included more specific codes for DSH, but only *ICD-9-CM* was available on CDARS. Also, the data captured patients who presented to the ED with DSH, but neglected those not admitted through the ED. Another limitation is the lack of consideration for other potential confounding variables, such as the changing social conditions in 2019. In addition, the use of linear interpolation to adjust population counts may introduce bias, as population changes during the pandemic could affect the results.

### Conclusion

The results indicate increasing adjusted DSH incidence ratios among males and individuals with alcohol and drug-related mental health conditions. This underscores the need for continued surveillance beyond the COVID-19 pandemic to determine if there is a spill-over effect. Importantly, all stakeholders—including policy makers, psychiatrists, and health care professionals—should collaborate to raise awareness and provide support for these vulnerable groups in Hong Kong. Targeted interventions and policies are essential to address the mental health challenges exacerbated by the pandemic and ensure better outcomes for those affected.

## Supplementary material

10.2196/57500 Multimedia Appendix 1 Additional tables.

10.2196/57500 Multimedia Appendix 2 Ethical approval.

10.2196/57500 Multimedia Appendix 3 The average observation room staying time between the years 2019 and 2022. The red highlight corresponds to the dip in the monthly deliberate self-harm–related emergency department visits seen in Figure 1.

10.2196/57500 Multimedia Appendix 4 The average total staying time between the years 2019 and 2022. The red highlight corresponds to the dip in the monthly deliberate self-harm–related emergency department visits seen in Figure 1.

10.2196/57500 Multimedia Appendix 5 The average cubicle time between the years 2019 and 2022. The red highlight corresponds to the dip in the monthly deliberate self-harm–related emergency department visits seen in Figure 1.

10.2196/57500 Multimedia Appendix 6 The average waiting time to triage between the years 2019 and 2022. The red highlight corresponds to the dip in the monthly deliberate self-harm–related emergency department visits seen in Figure 1.

## Abbreviations

AIC: Akaike Information Criterion
aIR: adjusted incidence ratio
CDARS: Clinical Data Analysis and Reporting System
DSH: deliberate self-harm
ED: emergency department
*ICD*: *International Classification of Diseases*
*ICD-9-CM*: *International Classification of Diseases, Ninth Revision, Clinical Modification*
ITS: interrupted time series
oIR: observed incidence ratio
