# Supplementary material for: Incidence of Deliberate Self-Harm in Hong Kong Before and During the COVID-19 Pandemic: Population-Wide Retrospective Cohort Study
Source: JMIR Public Health Surveill. 2025 Feb 10;11:e57500. doi: 10.2196/57500 (PMC11832357; doi:10.2196/57500)
Supplement: Multimedia Appendix 1 [file publichealth-v11-e57500-s001.docx]

**Table S1.** ICD-9-CM codes used to define mental health issues, mood disorders, neurosis, alcohol & drug-associated mental health conditions, other psychotic illnesses, and developmental disorders.

| Co-occurring mental health issues | ICD-9-CM^a^ |
| --- | --- |
| **All mental health issues** | 290-316 |
| **Mood disorders** | 296, 311 |
| **Neurosis** | 300, 308-309 |
| **Alcohol & drug-associated mental health conditions** | 291-292, 303-305 |
| **Other psychotic illnesses** | 290, 293-295, 297-298 |
| **Developmental disorders** | 299, 313-315 |

*^a^ ICD-9-CM: International Classification of Diseases, Ninth Revision, Clinical Modification*

**Table S2.** Definitions of Hong Kong triage categories.

| Triage categories | Definition |
| --- | --- |
| **Category I** | Critical cases that require immediate treatment. |
| **Category II** | Emergency cases that will be treated within 15 minutes. |
| **Category III** | Urgent cases that will be treated within 30 minutes. |

**Table S3.** The number of observed visits, observed incidence ratio, and adjusted incidence ratio in DSH-related ED visits between 2016-2022.

|  |  | Years | | | | | | | | | |
| --- | --- | --- | --- | --- | --- | --- | --- | --- | --- | --- | --- |
|  |  | 2016-2019 | 2020 | | | 2021 | | | 2022 | | |
|  |  | Observed Visits  [N, (%)] | oIR*^a^* | aIR*^b^* | *P*-Value | oIR | aIR | *P*-Value | oIR | aIR | *P*-Value |
| **Female Teenager (12-17 years old)** | | | | | | | | | | | |
|  | Mood disorders | 72 (29) | 2.25 | 0.93 | .67 | 4.99 | 1.43 | .12 | 4.36 | 0.87 | .70 |
|  | Neurosis | 72 (29) | 1.62 | 0.71 | .98 | 3.82 | 1.21 | .20 | 2.96 | 0.68 | .91 |
|  | Other psychotic illnesses | 7 (3) | 1.79 | 1.32 | .25 | 4.00 | 2.67 | .04 | 1.66 | 1.02 | .51 |
| **Male Teenager (12-17 years old)** | | | | | | | | | | | |
|  | Mood disorders | 11 (14) | 1.33 | 0.49 | .97 | 2.29 | 0.54 | .90 | 2.00 | 0.31 | .96 |
|  | Neurosis | 18 (23) | 1.07 | 0.98 | .55 | 3.04 | 2.73 | .009 | 2.31 | 2.06 | .09 |
|  | Other psychotic illnesses | 2 (2) | 1.71 | NA*^c^* | NA*^c^* | 1.14 | NA | NA*^c^* | 1.71 | NA | NA*^c^* |
| **Female Young adult (18-24 years old)** | | | | | | | | | | | |
|  | Mood disorders | 49 (17) | 1.73 | 1.25 | .10 | 1.82 | 1.15 | .30 | 2.57 | 1.41 | .14 |
|  | Neurosis | 66 (23) | 1.03 | 1.18 | .14 | 1.28 | 1.56 | .01 | 1.37 | 1.75 | .02 |
|  | Other psychotic illnesses | 7 (2) | 1.00 | 4.14 | .002 | 0.71 | 5.06 | .01 | 1.57 | 18.26 | .001 |
| **Male Young adult (18-24 years old)** | | | | | | | | | | | |
|  | Mood disorders | 13 (7) | 0.94 | 1.10 | .41 | 1.49 | 1.88 | .09 | 1.57 | 2.09 | .11 |
|  | Neurosis | 32 (18) | 0.63 | 0.89 | .65 | 0.85 | 1.39 | .16 | 0.72 | 1.34 | .26 |
|  | Other psychotic illnesses | 12 (7) | 0.58 | 0.95 | .54 | 0.75 | 1.49 | .19 | 1.00 | 2.38 | .06 |
| **Female Adult (25-64 years old)** | | | | | | | | | | | |
|  | Mood disorders | 237 (18) | 0.81 | 0.96 | .67 | 0.91 | 1.16 | .21 | 0.81 | 1.13 | .28 |
|  | Neurosis | 277 (21) | 0.88 | 1.11 | .10 | 0.90 | 1.28 | .01 | 0.95 | 1.51 | .001 |
|  | Other psychotic illnesses | 88 (7) | 0.89 | 1.20 | .04 | 1.02 | 1.59 | .003 | 1.03 | 1.85 | <.001 |
| **Male Adult (25-64 years old)** | | | | | | | | | | | |
|  | Mood disorders | 88 (7) | 0.94 | 1.46 | .006 | 1.19 | 2.22 | .001 | 1.23 | 2.73 | <.001 |
|  | Neurosis | 147 (11) | 0.93 | 1.45 | .002 | 1.30 | 2.44 | <.001 | 1.39 | 3.11 | <.001 |
|  | Other psychotic illnesses | 110 (9) | 0.81 | 1.22 | .11 | 1.38 | 2.50 | <.001 | 1.50 | 3.23 | <.001 |
| **Female Elderly (65-84 years old)** | | | | | | | | | | | |
|  | Mood disorders | 27 (21) | 1.19 | 1.51 | .05 | 1.26 | 1.77 | .05 | 1.26 | 1.96 | .08 |
|  | Neurosis | 20 (16) | 1.05 | 1.16 | .29 | 1.20 | 1.39 | .18 | 1.85 | 2.24 | .04 |
|  | Other psychotic illnesses | 12 (9) | 1.04 | 0.95 | .57 | 0.87 | 0.76 | .74 | 2.00 | 1.70 | .15 |
| **Male Elderly (65-84 years old)** | | | | | | | | | | | |
|  | Mood disorders | 14 (8) | 1.26 | 1.20 | .34 | 1.56 | 1.47 | .22 | 0.81 | 0.76 | .65 |
|  | Neurosis | 20 (11) | 0.97 | 1.01 | .46 | 1.23 | 1.30 | .24 | 1.79 | 1.95 | .09 |
|  | Other psychotic illnesses | 12 (7) | 1.30 | 1.48 | .13 | 2.26 | 2.72 | .02 | 2.70 | 3.45 | .03 |

*^a^ oIR: Observed Incidence Ratio*

*^b^ aIR: Adjusted Incidence Ratio*

*^c^ NA: Not applicable*
