# Supplementary material for: Incidence of Deliberate Self-Harm in Hong Kong Before and During the COVID-19 Pandemic: Population-Wide Retrospective Cohort Study
Source: JMIR Public Health Surveill. 2025 Feb 10;11:e57500. doi: 10.2196/57500 (PMC11832357; doi:10.2196/57500)
Supplement: Multimedia Appendix 2 [file publichealth-v11-e57500-s002.docx]

10^th^ January, 2025

Mengfei Sun

Production Editor

JMIR Publications

Dear Mingfei Sun,

Re: Statement of Ethics Review Board Approval

Manuscript Number: 57500

I hereby confirm that this study obtained ethical approval by The University of Hong Kong Institutional Review Board (UW 20-112).

Thank you for your attention.

Yours Sincerely,

Prof. Abraham K. C. Wai
