# Supplementary figures and images for: Incidence of Deliberate Self-Harm in Hong Kong Before and During the COVID-19 Pandemic: Population-Wide Retrospective Cohort Study
Source: JMIR Public Health Surveill. 2025 Feb 10;11:e57500. doi: 10.2196/57500 (PMC11832357; doi:10.2196/57500)

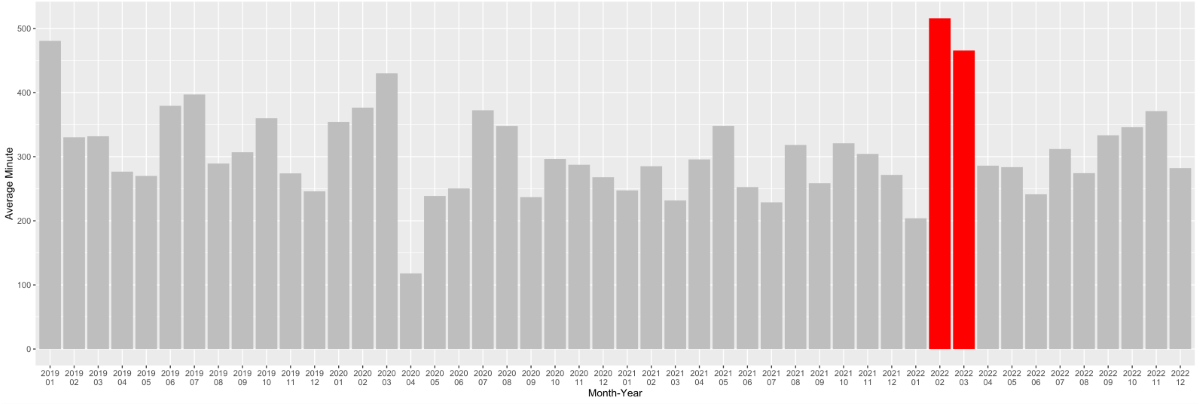

Supplement: Multimedia Appendix 3 [file publichealth-v11-e57500-s003.png]

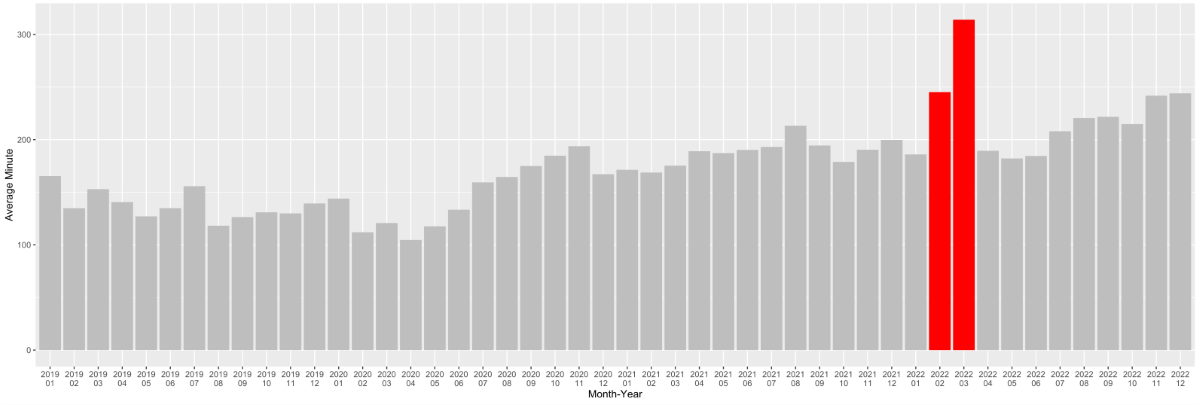

Supplement: Multimedia Appendix 4 [file publichealth-v11-e57500-s004.png]

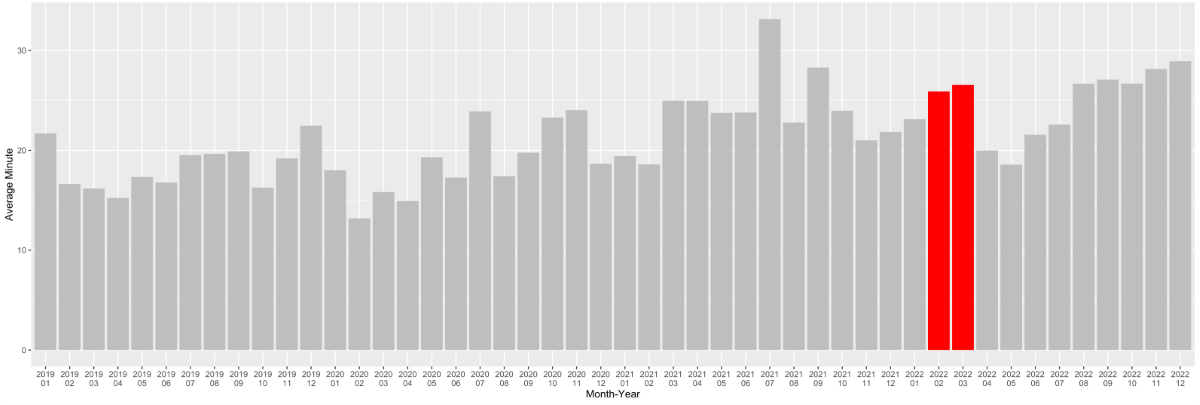

Supplement: Multimedia Appendix 5 [file publichealth-v11-e57500-s005.png]

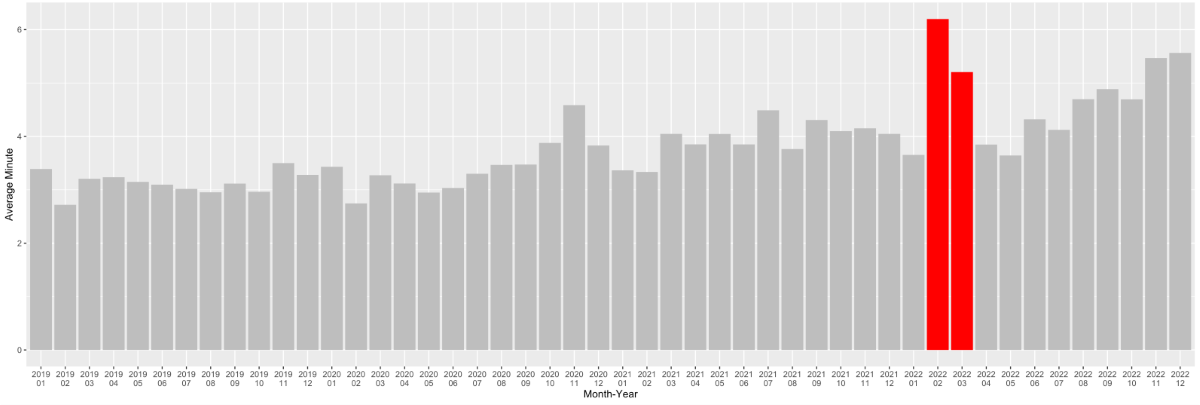

Supplement: Multimedia Appendix 6 [file publichealth-v11-e57500-s006.png]
